# Purinergic signal transduction and metabolic regulation by ENTPD5 and ENTPD6

**DOI:** 10.3389/fendo.2026.1680378

**Published:** 2026-01-28

**Authors:** Yihang Qi, Xuefei Li, Xinyu Song, Wenyi Wei, Ionita Ghiran, Simon C. Robson

**Affiliations:** 1 Center for Inflammation Research, Department of Anesthesia, Critical Care & Pain Medicine, Beth Israel Deaconess Medical Center, Harvard Medical School, Boston, MA, United States; 2 Department of Anesthesiology, Jiangsu Province Hospital of Chinese Medicine, Affiliated Hospital of Nanjing University of Chinese Medicine, Nanjing, Jiangsu, China; 3 Department of Medical Oncology, Qilu Hospital of Shandong University, Jinan, China; 4 Department of Pathology, Beth Israel Deaconess Medical Center, Harvard Medical School, Boston, MA, United States

**Keywords:** cancer, CD39L2, CD39L4, ectonucleoside triphosphate diphosphohydrolase 5, ectonucleoside triphosphate diphosphohydrolase 6, ENTPD5, ENTPD6, purinergic signaling pathway

## Abstract

ENTPD5 and ENTPD6 are members of the CD39-ectonucleoside triphosphate diphosphohydrolase (CD39-ENTPD) family, which play an important role in modulating the purinergic signaling pathway. Most of the knowledge in this area has been obtained by studying CD39/ENTPD1, the prototype member of this family, and evaluating the translational potential by either treating inflammation directly with recombinant proteins or by using antagonists to elicit immune responses in cancer. ENTPD5 and ENTPD6, “orphan-type” ectonucleotidases, are understudied to date, although both are expressed at high levels in various tissues, where they appear involved in regulating signal transduction, cellular energy, and metabolism. ENTPD5 is abnormally overexpressed in several types of malignancies, including prostate, liver, lung, and ovarian cancers. ENTPD5 appears to promote protein glycosylation and folding in part by regulating UDP and UMP levels, thereby enhancing the survival and proliferation of somatic or cancer cells. As such, ENTPD5 has been considered a potential proto-oncogene and a therapeutic target in cancer treatment. In contrast, despite comparable functionality, the related ENTPD6 shows relatively stable expression across tissues in both normal and pathological conditions, with specific roles in cancer yet unclear. This review provides a comprehensive overview of these two understudied ectoenzymes, detailing their shared molecular structures and control of purinergic signal transduction. In addition, we explore different patterns of tissue and organelle expression of these ecto-enzymes and propose relevance to the modulation of cellular metabolism, as would be important in cancer. We review the sometimes conflicting evidence from experimental animal models and propose potential future clinical applications. This review offers insights into the roles of this distinct duo of ENTPD family members to support future basic and translational research in this field.

## 1 Introduction

The ectonucleoside triphosphate diphosphohydrolases (ENTPDases) are a family of ectoenzymes, responsible for hydrolyzing extracellular nucleoside diphosphates and triphosphates. This family includes eight closely related members that exist in multiple forms, suggesting they originated through gene duplication. These ENTPDases are either integral plasma membrane-bound ectoenzymes or predominantly expressed within the endoplasmic reticulum (ER) and Golgi apparatus; although some of these proteins can also be secreted into the extracellular fluid. These family members exhibit distinct expression patterns and subcellular localizations, which likely influence their functional specialization.

Among the eight known human NTPDase isoforms, ENTPD5 (ectonucleoside triphosphate diphosphohydrolase 5) and ENTPD6 (ectonucleoside triphosphate diphosphohydrolase 6) are the ones that face the luminal side of the endoplasmic reticulum (ER) and Golgi apparatus, respectively. Specifically, ENTPD5 and ENTPD6 are thought to participate in glycosylation-related nucleotide processing by regulating levels of UDP and nucleotide sugars (1, 2). Both enzymes can also be released in a soluble form, and they exhibit substrate specificity for nucleoside diphosphates such as UDP and GDP. This highlights their potential roles in regulating intracellular nucleotide pools during glycoprotein maturation (3).

ENTPD5 is predominantly localized to the ER, where it operates as a uridine diphosphatase (UDPase). Although ENTPD5 lacks transmembrane domains, an N-terminal signal peptide enables its retention within the ER lumen. It may also be secreted in a soluble form (4). ENTPD5 hydrolyzes UDP to UMP, enabling UDP-glucose transport into the ER lumen and maintaining the calnexin–calreticulin cycle for efficient N-glycosylation (5). ENTPD5 is notably overexpressed in cancer, where it contributes to ER homeostasis, protein folding, and cell survival, particularly in tumors driven by aberrant activation of P13K/AKT or mutant p53 signalling (6, 7).

In contrast, ENTPD6 is localized primarily to the Golgi apparatus and plasma membrane. It exists in both membrane-bound and soluble forms, the latter resulting from proteolytic cleavage of the signal peptide. Structurally, ENTPD6 possesses two transmembrane domains flanking an outward-facing catalytic site. ENTPD6 exhibits low specificity for monophosphates or triphosphates but high specificity for nucleoside diphosphates (NDPs) such as GDP, IDP, and UDP (8). Although ENTPD6 does not participate directly in ER proteostasis, it has been thought to be involved in localized extracellular nucleotide hydrolysis and purinergic tone modulation, particularly in neuroendocrine and reproductive tissues.

Hence, ENTPD5 and ENTPD6 operate in different organelles: the ER and the Golgi apparatus. However, both ecto-enzymes preferentially hydrolyze nucleoside diphosphates and contribute to the regulation of nucleotide homeostasis within the secretory pathway. These shared biochemical mechanisms indicate a common role in supporting glycoprotein maturation and maintaining biosynthetic efficiency across secretory compartments. Furthermore, the shared substrate preference of ENTPD5 and ENTPD6 for NDPs is similar to the ligand specificity of P2Y receptors, particularly those activated by UDP and ADP.

ENTPD5 and ENTPD6 may influence the local availability of nucleotide ligands and modulate the activation thresholds and signaling pathways of various G protein–coupled receptors, such as P2Y6 and P2Y14. Furthermore, ENTPD5 and ENTPD6 may play a broader role in linking nucleotide metabolism with purinergic signal transduction, particularly in secretory and immune-responsive cells (9).

CD39 (ENTPD1) was the first of these family members to be extensively studied and has an immunomodulatory role in cancer and inflammation. Work on CD39 has resulted in the development of innovative therapeutic strategies, including immune checkpoint inhibition and immunometabolic modulation. These studies identified CD39 and CD73 (Cluster of Differentiation 73/Ecto-5′-nucleotidase) as novel targets for cancer immunotherapy by modulating extracellular nucleotide pools and immune checkpoint microenvironments (10). In addition, other researchers and we have highlighted the clinical importance of purinergic signaling pathways, including CD39-mediated ATP phosphohydrolysis, in the context of inflammation, ischemia-reperfusion injury, and immune regulation (11).

Overall, dysregulation of ENTPDases may disrupt nucleotide-sensitive signaling cascades and metabolic balance, potentially contributing to diseases such as cancer, metabolic syndromes, and immune system dysfunction. The specific localization and activity of ENTPD5 and ENTPD6 serve as bridges linking biosynthetic demands, purinergic signaling, and cellular adaptation processes. In this review, we focused on these two members in the ENTPD family, where the prototype was recognized as CD39/ENTPD1. The central role of CD39 in purinergic biology has been well described by members of our laboratory and many others after its discovery in 1996 (12). Possible applications of CD39 as a checkpoint inhibitor are now being tested in clinical trials (10).

**Figure 1:**
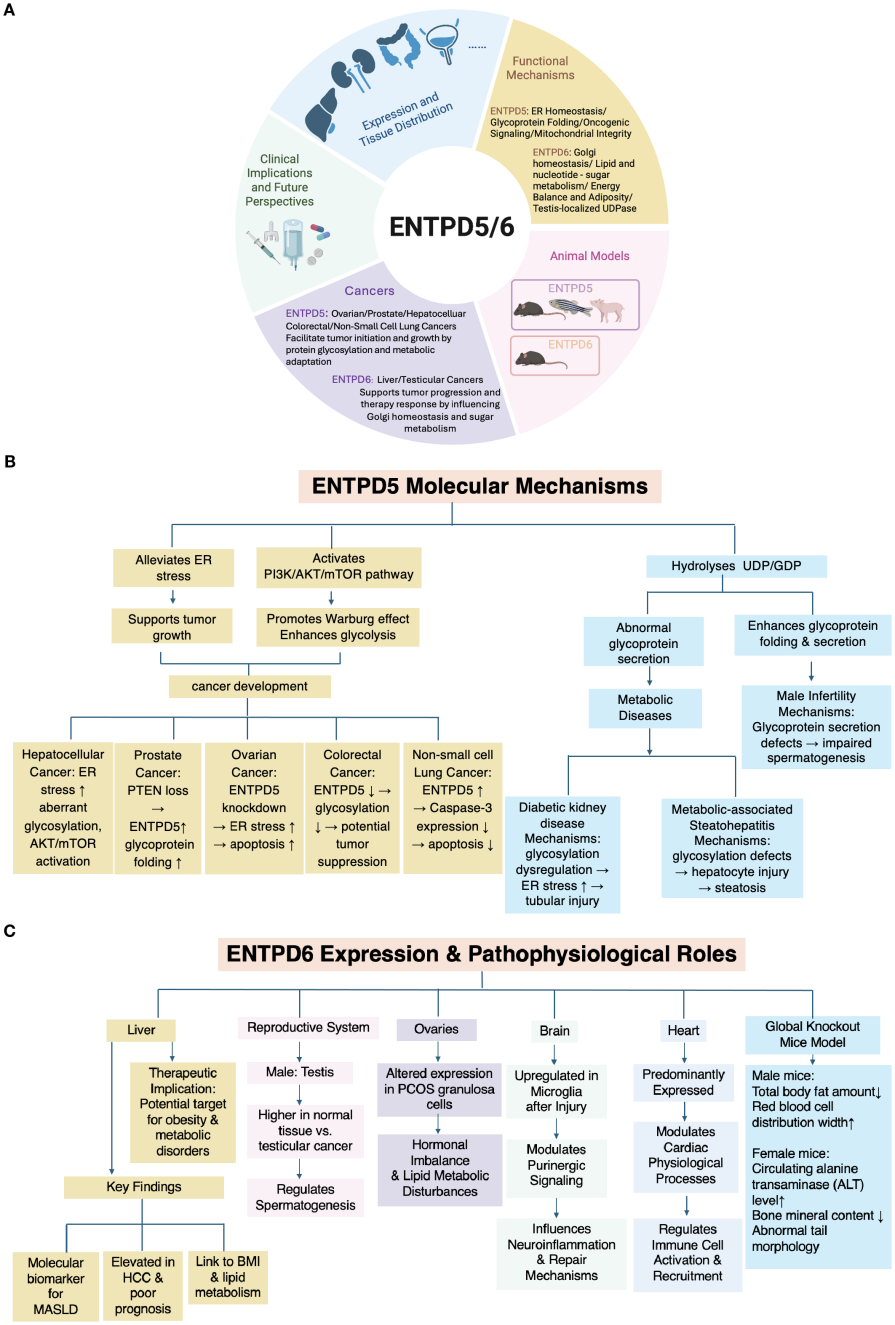
**(A)** Schematic overview of expression, functional mechanisms, animal models, tumorigenesis, and potential clinical implications of ENTPD5 and ENTPD6 (created with BioRender.com). **(B)** Molecular mechanisms of ENTPD5. **(C)** Expression and pathophysiological roles of ENTPD6.

Since ENTPD5 and ENTPD6 are likely duplications of the primordial gene, these ectoenzymes do share some similarities with the other members, but are also quite distinct from these and CD39. We have summarized current animal models, potential mechanisms in cancer development, and ongoing and future potential clinical applications in detail to give a more comprehensive picture to assist readers in better understanding their roles and potential drug-development directions. (**Figure 1A**).

## 2 Expression and tissue distribution of ENTPD5 and ENTPD6

### 2.1 ENTPD5 expression in normal tissues

ENTPD5 is predominantly expressed in the liver, where it plays a crucial role in hydrolyzing extracellular ATP into ADP, thus helping to regulate metabolic balance (13). Notably, a decrease in ENTPD5 levels is strongly linked to the development of non-alcoholic fatty liver disease (NAFLD) (13). In studies involving both steatotic mouse models and human liver samples, lower expression of ENTPD5 has been shown to increase hepatic gluconeogenesis and lipid accumulation. This increase subsequently worsens insulin resistance and contributes to obesity (13). The mechanism behind this involves the ADP produced by ENTPD5-mediated ATP hydrolysis. This ADP activates the purinergic receptor P2Y12, and activation of this receptor subsequently inhibits gluconeogenesis and lipid accumulation in liver cells. Additionally, this pathway decreases the expression of adrenomedullin (ADM), which is important for promoting thermogenesis in brown adipose tissue (BAT) and enhancing overall energy metabolism in the body (13).

In obese mouse models, upregulating ENTPD5 expression in hepatocytes led to significant improvements in glucose tolerance and lipid metabolism, while also reducing weight gain. Conversely, knocking down ENTPD5 resulted in increased gluconeogenesis, enhanced lipid accumulation, worsened insulin resistance, and greater weight gain (13). The active expression of ENTPD5 in hepatocytes is further supported by transcriptomic datasets and *in situ* hybridization, emphasizing its significant physiological role in basal hepatic metabolism (14).

In the skeletal system, ENTPD5 is specifically found in osteoblasts, and genetic screening in zebrafish has shown that the absence of ENTPD5 disrupts skeletal mineralization. ENTPD5 works in conjunction with ectonucleotide pyrophosphatase phosphodiesterase 1 (ENPP1) to regulate the balance between phosphate and pyrophosphate, which is essential for normal bone development and mineralization (15).

In the kidney, ENTPD5 is predominantly expressed at high levels in proximal tubular epithelial cells, where it influences the rate of ER protein N-glycosylation and impacts cellular processes such as proliferation and differentiation. The overexpression of ENTPD5 has been found to reduce ER stress and encourage compensatory cellular proliferation, resulting in tubular hypertrophy (16). These findings highlight its key role in maintaining renal tubular homeostasis. A summary of expression and molecular mechanisms of ENTPD5 is shown in **Figure 1B**.

### 2.2 ENTPD6 expression varies across tissues

ENTPD6 shows distinct patterns of expression across various tissues, with notable levels found in the liver, reproductive organs, brain, and heart, where the ectoenzyme plays a role in regulating various pathophysiological processes (2, 17, 18).

In the liver, ENTPD6 has been identified as a crucial molecular biomarker for metabolic dysfunction-associated steatotic liver disease (MASLD), with elevated expression linked to the development and progression of hepatocellular carcinoma (HCC) and poor patient outcomes (19). Large-scale genome-wide association studies (GWAS) have also indicated that variants of the ENTPD6 gene are significantly associated with body mass index (BMI) (20), highlighting its involvement in energy balance and lipid metabolism pathways, which positions it as a potential therapeutic target for obesity and related metabolic disorders.

In the reproductive system, ENTPD6 is prominently expressed in normal testicular tissue, showing higher levels in non-neoplastic tissue compared to testicular cancer tissue, and particularly elevated in seminomas among testicular tumor subtypes (21). Clinical studies have noted significant upregulation of ENTPD6 in testicular tissue from patients with azoospermia, and its genomic location aligns with common chromosomal breakpoint regions (22), suggesting a regulatory role in spermatogenesis-related gene networks. In female reproductive health, significant changes in ENTPD6 expression have been observed in ovarian granulosa cells from patients with polycystic ovary syndrome (PCOS) (23), correlating with hormonal imbalances and lipid metabolic disturbances, thereby emphasizing its critical regulatory role in both male and female reproductive health.

In brain tissue, the expression of ENTPD6 varies in response to different types of injury models, such as traumatic and ischemic injuries (24). For example, following brain injury, there is an increase in ENTPD6 protein expression in microglia, which may influence purinergic signaling pathways and microglial responses to cellular damage (24). This increase underscores the dynamic nature of ENTPD6 expression within the nervous system and suggests a role in inflammation and repair mechanisms.

Studies indicate that ENTPD6, also referred to as CD39L2, is predominantly expressed in the heart (25), which suggests it plays a significant role in local purinergic signaling within cardiac tissue. This expression highlights its potential involvement in various cardiac physiological processes, as well as in the regulation of immune cell activation and recruitment. A summary of expression and pathophysiological roles of ENTPD6 is shown in **Figure 1C**.

A summary of differential patterns of ENTPD5 and ENTPD6 expression across tissues is shown in **Table 1**. The distinct expression profiles of ENTPD5 and ENTPD6 suggest not only tissue-specific glycoprotein processing and nucleotide turnover between the two enzymes but also distinct regulatory pathways and stress-responsive adaptations. These features position ENTPD5 and ENTPD6 as attractive candidates for further exploration in the context of metabolic regulation, cellular stress responses, and tissue-specific nucleotide signaling.

**Table 1:** Tissue-specific expression pattern of ENTPD5 and ENTPD6.

Tissue/Cell type	ENTPD5 expression & function	ENTPD6 expression & function
Liver	High in hepatocytes; inhibits gluconeogenesis/lipid accumulation; protects against NAFLD/MASLD.	Upregulated in MASLD/HCC; genetic variants associated with BMI; role in lipid metabolism and cancer progression.
Skeletal System	Localized to osteoblasts; essential for bone mineralization.	Not prominently reported.
Kidney	High in proximal tubular epithelial cells; mitigates ER stress, promotes tubular hypertrophy.	Not prominently reported.
Reproductive System	Not prominently reported.	High in testis (non-neoplastic > cancerous); altered in azoospermia and PCOS granulosa cells; regulates spermatogenesis and hormonal/lipid metabolism.
Brain	Not prominently reported.	Induced in microglia upon injury; modulates purinergic signaling and neuroinflammation.
Heart	Not prominently reported.	Prominently expressed; involved in cardiac purinergic signaling and immune regulation.

## 3 Functional mechanisms of ENTPD5 and ENTPD6

### 3.1 A potential enzymatic nexus linking nucleotide metabolism, glycosylation, and immune regulation

**Figure 2:**
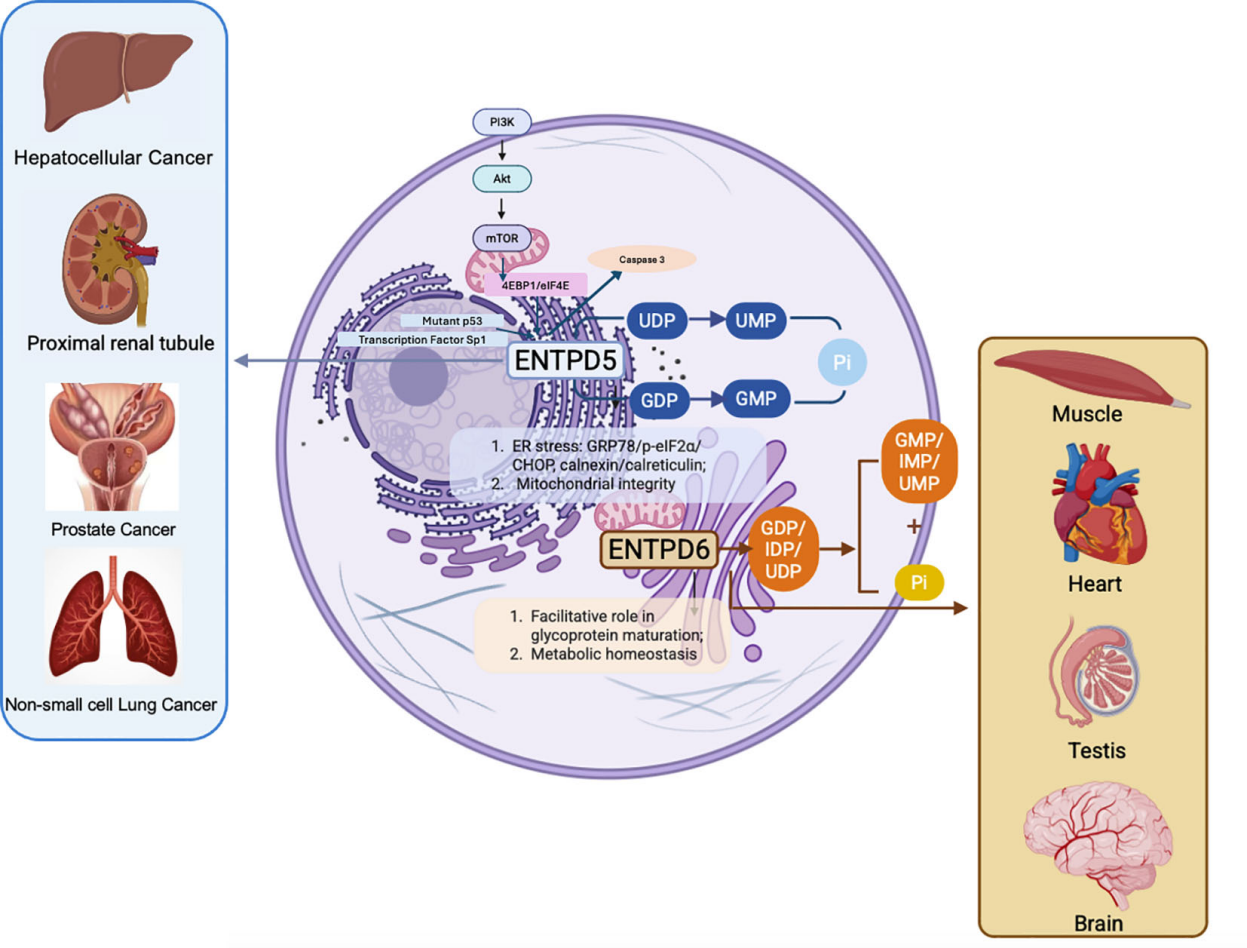
Role of ENTPD5 and ENTPD6 in cellular processes, expression patterns and association with tumorigenesis pathways (created with BioRender.com).

ENTPD5 and ENTPD6 are two structurally related UDPases that localize to intracellular compartments and regulate nucleotide metabolism, protein glycosylation, and stress adaptation. Both ENTPD5 and ENTPD6 catalyze the hydrolysis of NDPs in a calcium- or magnesium-dependent manner, but they exhibit distinct substrate preferences and subcellular localizations (**Figure 2**).

ENTPD6 preferentially hydrolyzes GDP, IDP, and UDP over other nucleotides such as CDP, ADP, GTP, CTP, UTP, and ATP. Similarly, ENTPD5 preferentially hydrolyzes GDP, IDP, and UDP over ADP and CDP. These selective substrate profiles correspond to their distinct biological roles: ENTPD6 may largely facilitate the exchange of UDP-sugars in the Golgi apparatus, whereas ENTPD5 may have comparable and other roles in the endoplasmic reticulum, detailed below.

The relationship between nucleotide-metabolizing enzymes and purinergic receptors may be a critical determinant of cell fate in inflammatory and metabolic pathways. P2Y6 and P2Y14-G protein–coupled receptors activated by UDP and UDP-sugars, respectively, are expressed in various immune and epithelial cells. In these cells, they regulate chemotaxis, cytokine production, and barrier function. In colorectal cancer (CRC), elevated extracellular UDP levels are proposed to promote tumor progression via sustained activation of P2Y6 signaling (26). Similarly, the UDP–cytidine diphosphate–adenosine (UDP–CDA)–P2Y6 signaling axis has been shown to drive M2-type macrophage polarization, creating an immunosuppressive environment conducive to the progression of pancreatic ductal adenocarcinoma (PDAC) (27). Notably, the significant correlation between the UDP-glucose-specific G protein–coupled receptor P2Y14 and clinical outcomes in patients with head and neck squamous cell carcinoma (HNSC) suggests a broader role for UDP-sugar–sensing purinergic pathways in shaping immune responses and influencing cancer prognosis (28).

The substrate specificity of ENTPD5 and ENTPD6 suggests a potential upstream regulatory role in the pathway involving UDP and UDP-sugars as ligands and the P2Y6 and P2Y14 receptors. This biochemical axis of linking intracellular nucleotide metabolism with extracellular purinergic signaling may represent a novel connection between glycosylation regulation, specifically N-linked glycosylation, and immune modulation in cancer and inflammation.

### 3.2 ENTPD5 upregulation by mutant p53 promotes cancer metastasis

Mutant p53 is one of the most common genetic changes found in human cancers. This mutation not only results in the loss of its tumor-suppressing abilities but also allows it to gain oncogenic properties that promote cancer progression. Research has shown that ENTPD5 is a direct target of mutant p53; specifically, mutant p53 interacts with the transcription factor Sp1 to enhance the expression of ENTPD5 (29). ENTPD5 is a UDPase located in the endoplasmic reticulum (ER), which plays a crucial role in the proper folding of N-glycoproteins by influencing nucleotide-dependent steps in glycosylation. This process is vital for the maturation of glycoproteins on the cell surface, which in turn supports cancer cell migration, invasion, and metastasis (29, 30). Timofeev and colleagues further illustrated that mutant p53 increases ENTPD5 levels, thereby boosting the protein folding capacity within the ER (30). This increase significantly enhances the metastatic potential in various cancer models, establishing ENTPD5 as an important factor in the tumor progression driven by mutant p53.

Additionally, a comprehensive analysis by Vogiatzi and others confirmed a strong link between p53 gain-of-function mutations and ENTPD5 expression (30), emphasizing the role of ENTPD5 in the folding of N-glycoproteins as a key mechanism in tissue remodeling and the spread of cancer to the lungs. *In vivo* studies have further supported the significance of ENTPD5 in tumor clonogenicity, structural changes, and the spread of cancer, highlighting its promise as a target for new therapeutic strategies.

### 3.3 Hepatic ENTPD5 in glucose-lipid metabolism and thermogenesis

ENTPD5 is predominantly expressed in hepatocytes and shows decreased levels in fatty liver models. Research by Ma et al. demonstrated that hepatic ENTPD5 plays a crucial role in hydrolyzing extracellular ATP to ADP (13), which subsequently activates the P2Y12 receptor. This activation leads to a reduction in gluconeogenesis and lipid accumulation. Additionally, ENTPD5 is involved in inhibiting the secretion of ADM, a peptide hormone that reduces UCP1 expression in BAT, thus promoting thermogenesis. In studies involving obese mice, overexpression of ENTPD5 improved metabolic abnormalities, while its knockdown worsened metabolic dysregulation, highlighting its essential role in maintaining systemic energy balance (13). Furthermore, clinical findings reinforce the significance of this pathway - reduced hepatic ENTPD5 expression in patients with NAFLD is associated with increased serum ADM levels and higher BMI (13). This correlation suggests that ENTPD5 might serve as both a biomarker and a potential therapeutic target for metabolic diseases.

### 3.4 ENTPD5 is a nodal regulator of oncogenic signaling, metabolic reprogramming, and organellar homeostasis

Under oncogenic conditions such as PTEN loss and PI3K/AKT/mTOR activation, cancer cells exhibit heightened protein synthesis and N-glycosylation stress, increasing their reliance on ER function (7). ENTPD5 is transcriptionally upregulated in this context and serves as a critical effector linking oncogenic signaling to N-glycosylation fidelity. Its activity supports a futile ATP hydrolysis cycle that, although energetically wasteful, enhances glycolytic flux and promotes the Warburg effect. In contrast, ENTPD5 loss impairs glycoprotein folding, suppresses glycolysis, and inhibits tumor growth. Moreover, ENTPD5 is regulated by HRD1 (HMG-CoA Reductase Degradation Protein 1)-mediated ER-associated degradation, highlighting its role in proteotoxic stress adaptation (31). In AKT-driven tumors, ENTPD5 helps alleviate ER stress and sustains growth under metabolic burden (32). Beyond glycosylation, ENTPD5 also influences mitochondrial integrity. ENTPD5 depletion models in kidney disease show reduced mitochondrial abundance and increased apoptosis (16), suggesting a role in preserving organelle function through glycosylation quality control and ER-mitochondrial crosstalk.

### 3.5 ENTPD6 as a putative regulator of energy balance, adiposity, and immunity

Emerging evidence supports the possible involvement of ENTPD6 in metabolic homeostasis. ENTPD6 was found to be located at loci associated with obesity and type 2 diabetes risk, suggesting ENTPD6 plays a possible role in regulating central energy sensing and hepatic nucleotide metabolism, and elevated ENTPD6 levels may increase the risk of visceral obesity (33). Additionally, ENTPD6 was identified as a causal molecular marker for metabolic function, and it showed a causal association with metabolic dysfunction–associated steatotic liver disease (19). GWAS and gene set enrichment analyses conducted by Turcot et al. have identified genetic associations between ENTPD6 variants and BMI. ENTPD6 may affect energy intake or consumption and is related to central adiposity (20). In summary, ENTPD6 is associated with the risk of fatty liver, liver cancer, diabetes, and visceral obesity. It may also play a role in regulating energy metabolism pathways. However, it is still not clear whether ENTPD6 acts upstream (driving metabolic effects) or downstream (responding to altered metabolism), which will need further studies. ENTPD6 has also been proposed as a molecular biomarker for MASLD and is elevated in hepatocellular carcinoma (HCC), correlating with poorer clinical outcomes.

Forti et al. found that ENTPD6 is highly expressed in HNSCCs, and P2Y2R is the primary P2 receptor with significant functional activity. The MOC2 tumors in the wild-type mice were larger, where fewer tumor-infiltrating “CD3+CD4+IFNγ+ T cells” were found when compared to tumors in P2Y2R^-/-^mice. These findings suggest that ENTPD6 may regulate the availability of extracellular nucleotides such as ATP and UTP, thereby sustaining P2Y2R activation and indirectly facilitating tumor occurrence and development in HNSCC (34).

“Beyond the observed functionality in modulating hepatic metabolism, ENTPD6 exhibits tissue-specific expression and putatively diverse other primary roles across a range of other organ and disease systems. In the reproductive system, ENTPD6 is highly expressed in the testis, where it has been implicated in the regulation of spermatogenesis; notably, expression is decreased in testicular cancer relative to normal testicular tissues (21). In females, altered ENTPD6 expression has been reported in the ovarian granulosa cells from patients with PCOS (23), which is associated with hormonal imbalance and lipid metabolic disturbances. ENTPD6 is also expressed in the central nervous system (24), where it is upregulated in microglia following injury. In this context, ENTPD6 modulates purinergic signaling and influences neuroinflammatory responses and repair mechanisms. Lastly, in the cardiovascular system, ENTPD6 has been shown to modulate cardiac pathophysiological processes and appears to regulate immune cell activation and recruitment (25).”

## 4 Animal models

### 4.1 *Entpd5* knockout mouse model

Read et al. developed a mouse model with *ENTPD5* deletion (*Entpd5*⁻/⁻) to study the physiological functions of ENTPD5 (14). These homozygous *Entpd5*⁻/⁻ mice appeared phenotypically normal at birth but later exhibited postnatal growth delay and further developed progressive hepatic diseases with age. Histopathological analyses revealed earlier hypertrophy of centrilobular hepatocytes, increased cell proliferation, and degeneration. Paradoxically, elderly mice developed spontaneous hepatocellular adenomas and carcinomas.

Furthermore, elevated cytochrome P450 enzyme activities in *Entpd5*⁻/⁻ mice, especially that of CYP1A and CYP3A isoforms, suggested hepatic adaptation to cellular injury. This study provided evidence that loss of ENTPD5 promotes chronic liver injury, compensatory proliferation, and hepatocarcinogenesis.

Similar pathological features were also observed in *ENTPD1*-deficient mice. According to Sun et al., the absence of CD39 (ENTPD1) resulted in elevated levels of extracellular ATP, subsequently activating hepatocyte proliferation pathways involving Ras-MAPK (Mitogen-activated protein kinase) and PI3K-mTOR (Phosphoinositide 3-Kinase -mechanistic Target of Rapamycin) signaling and inhibiting autophagy. As a result, cellular metabolism and persistent cell proliferation were disrupted. In both chemically induced and spontaneous models of hepatocellular carcinoma, *CD39*-null mice developed a significantly greater tumor burden and exhibited more aggressive tumor phenotypes. These findings provided evidence linking *ENTPD1* deficiency to the development of liver cancer under chronic inflammatory conditions (35).

These knockout models represented valuable tools for dissecting the molecular/purinergic pathways linking cell metabolism, nucleotide phosphohydrolysis, ER function, and HCC development, and could facilitate the preclinical evaluation of therapeutic interventions targeting these processes.

To further explore the physiological role of ENTPD5 in metabolic liver diseases, as in hepatic steatosis, Yu et al. investigated its expression and functional significance in a high-fat, high-carbohydrate (HFHC) diet-induced mouse model of metabolism-associated steatohepatitis (MASH) (36). Through integrated transcriptomic analyses and machine learning-based gene selection from human liver samples, ENTPD5 was identified as one of seven glycosylation-related hub genes linked to obesity and poor responses to weight loss interventions.

The upregulation of *Entpd5* was subsequently validated in the livers of HFHC-fed obese mice, which exhibited pronounced hepatic steatosis, fibrosis, and increased serum triglyceride levels. Quantitative PCR confirmed a significant elevation of *Entpd5* mRNA expression in MASH model mice, which was consistent with observations in MASH patient liver samples. Functional enrichment and gene co-expression analyses indicated close involvement of ENTPD5 in ER stress, protein glycosylation, and mitochondrial metabolic pathways—processes known to contribute to hepatocellular injury in metabolic disorders.

These and other studies highlight the utility of the HFHC-fed and other mouse studies as *in vivo* models for studying ENTPD5-related mechanisms in MASH and fatty liver pathogenesis. Parenthetically, other metabolic experimental models studying the induction of obesity in mutant mice with global *ENTPD3* deletion indicated paradoxical resistance to obesity-associated glucose intolerance, likely mediated through metabolic changes in adipocytes (9). These studies supported the future potential of ENTPD members as therapeutic targets for obesity-associated liver disease, but also indicated the likely complexity of the underlying cell-specific responses to purinergic modulation.

Studies of 7,12-dimethylbenzanthracene (DMBA) triggering of ENTPD5 upregulation also show relationships to the development of mammary cancer. Solanas et al. investigated the expression of the proto-oncogenic form of ENTPD5 (also known as in several publications as PCPH, Prostate Cancer Progression-Associated Protein) in a chemically induced rat model of mammary carcinogenesis (37). Female Sprague-Dawley rats were administered a single dose of DMBA, resulting in the development of both benign and malignant mammary tumors. Western blot analysis of tumor and normal mammary tissues demonstrated that PCPH expression was deregulated during tumorigenesis. While normal mammary epithelium predominantly expressed a 27-kDa PCPH polypeptide, this form was significantly downregulated or absent in tumors. In contrast, the 47-kDa variant was frequently upregulated in both benign and malignant lesions. Many tumors also expressed additional atypical PCPH isoforms not found in normal tissues, indicating a complex alteration of PCPH protein processing or splicing. Immunohistochemical staining further confirmed reduced or absent PCPH signal in neoplastic tissues.

These findings suggest that PCPH/Entpd5 dysregulation is an early and persistent molecular event in DMBA-induced mammary tumor development. This rat model not only mirrors features of human breast carcinogenesis but also provides a relevant *in vivo* experimental system to study the tissue-specific regulation and functional consequences of ENTPD5/PCPH expression during tumor progression.

### 4.2 ENTPD5 zebra fish model

In a zebrafish forward genetic screen by Huitema et al., ENTPD5 was identified as an important factor for bone mineralization (15). The “*no bone* (nob) mutant”, which carries a loss-of-function mutation in the *Entpd5* gene, results in loss of mineralized bone structures during development, while the cartilage formation and osteoblasts are normal. This suggests that ENTPD5 is involved in the mineralization process but not in osteoblast differentiation or matrix deposition. Rescue experiments demonstrated that expression of wild-type *Entpd5* can restore skeletal mineralization in “*nob* mutants”. Normal mineralization could be reestablished even when *Entpd5* was expressed outside of osteoblasts, which indicates that ENTPD5 plays a potential role in regulating phosphate/pyrophosphate availability independently of osteoblasts. Additionally, “*nob* mutants” also showed impaired phosphate regulation. Downregulation of *Fgf23* and upregulation of the phosphate transporter *Npt2a* can be partially rescued by exogenous supplementation of phosphate.

Moreover, genetic interaction studies revealed that double mutants of *Entpd5* and *Enpp* (encoding an enzyme responsible for generating pyrophosphate, which inhibits the mineralization process) can restore normal skeletal mineralization. These findings make zebrafish *Entpd5* mutants a good model for studying the phosphate-dependent mineralization process and provide valuable insights into the molecular regulations.

Using the zebrafish model, Suarez-Bregua et al. studied the relationship between neuron activity and skeletal mineralization in phosphate-related pathways (38). They investigated changes in gene expression following selective two-photon laser ablation of Pth4:eGFP-expressing hypothalamic neurons, a newly identified neuroendocrine population involved in brain–bone communication. Early disruption of these neurons led to significant impairment in craniofacial bone mineralization, while cartilage development remained unaffected. This phenotype was associated with downregulation of *Entpd5* and *Phex*, while the expression levels of phosphate regulators such as *Fgf23*, *Npt2a*, and *Npt2b* were unchanged. The observed decrease in *Entpd5* suggests a role in maintaining adequate phosphate availability for normal skeletal mineralization. Additionally, reduced expression of osteoblast differentiation markers—including delayed *Sp7* expression at 3 dpf and decreased *Sparc* expression at 7 dpf—was observed in ablated larvae, indicating that loss of Pth4-expressing neurons impacts osteoblast maturation and bone matrix formation.

### 4.3 ENTPD5 swine model

Wu et al. established a diabetic nephropathy model in Guizhou miniature pigs by intraperitoneal injection of streptozotocin and evaluated the therapeutic effects of *Persicaria capitata* through dietary supplementation. After 16 weeks, treated pigs exhibited significant improvements in disease-related indices and renal histopathology.

The presence of *ENTPD5* at both transcript and protein levels suggested a potential role in the renal-protective effects. The study also identified several metabolites and microbial taxa associated with treatment, indicating potential host-microbe interactions. This pig model offers valuable insight into the relevance of *ENTPD5* in large-animal studies of diabetic kidney disease and traditional medicine interventions (39).

### 4.4 *ENTPD6* knockout mouse model

Based on data from MGI (https://www.informatics.jax.org/allele/allgenoviews/

MGI:4363445), the ENTPD6 knockout mouse model exhibits sex-specific phenotypic traits. In male mice, a decrease in total body fat amount and an increase in red blood cell distribution width were observed. In female mice, increased circulating alanine transaminase (ALT) levels, abnormal tail morphology, and decreased bone mineral content were observed. However, the model did not show any lethal or significant growth and developmental defects. These findings suggest that ENTPD6 may play a gender-dependent role in fat metabolism, hematopoiesis, liver function, and bone development.

## 5 ENTPD5 and ENTPD6 in cancer

ENTPD5 and ENTPD6 exhibit distinct profiles in cancer biology. ENTPD5 has been identified as a proto-oncogene in several cancer types, including prostate cancer, hepatocellular carcinoma, and colorectal cancer. In addition, overexpression of ENTPD5 appears to support tumor growth by enhancing protein glycosylation, promoting metabolic adaptation, and facilitating protein folding and secretion.

In contrast, any potential role for ENTPD6 in cancer pathogenesis is less understood. However, some studies showed that ENTPD6 is potentially involved in tumor sensitivity to cancer therapies. Moreover, its potential involvement in extracellular nucleotide metabolism may influence protein glycosylation and potentially tumor behavior and progression.

### 5.1 ENTPD5 in cancer: a potential proto-oncogenic role

ENTPD5 has been identified as a proto-oncogene in several cancers, primarily due to its role in protein glycosylation, metabolic adaptation, and tumor cell survival. (**Table 2**).

**Table 2:** Emerging roles of ENTPD5 and ENTPD6 in cancer: molecular mechanisms.

NTPDase or ENTPD Family Member	Cancer type	Role	Mechanisms	Reference
ENTPD5	Ovarian Cancer	ENTPD5 knockdown induced endoplasmic reticulum stress and triggered cell apoptosis.	ENTPD5 knockdown activated the GRP78 (glucose-regulated protein 78)/p-eIF-2α (phosphorylated eukaryotic translation initiation factor 2 alpha)/CHOP (C/EBP homologous protein) pathway, which induced endoplasmic reticulum stress and triggered cell apoptosis.	Chen, X. et al. Knockdown of ENTPD5 inhibits tumor metastasis and growth via regulating the GRP78/p-eIF-2α/CHOP pathway in serous ovarian cancer (40).
		Overexpression of ENTPD5 is related to poor prognosis in ovarian cancer patients.	ENTPD5 was found significantly overexpressed in epithelial ovarian cancer tissues compared to normal ovarian tissues. Elevated ENTPD5 levels were significantly associated with reduced patient survival. ENTPD5 was highly enriched in several pathways related to metabolism and signal transduction, including WNT and insulin signaling.	Wang, H. et al. [ENTPD5 gene is highly expressed in epithelial ovarian cancer: analysis based on Oncomine database and bioinformatics] (41).
	Prostate Cancer	Overexpression of ENTPD5 is related to poor prognosis.	High ENTPD5 expression is closely associated with poor prognosis in prostate cancer patients.	Villar, J. et al. PCPH/ENTPD5 expression enhances the invasiveness of human prostate cancer cells by a protein kinase C delta-dependent mechanism (42).
		Expression of ENTPD5 contributes to cisplatin resistance.	Expression of PCPH, and its oncogenic variant mt-PCPH, contributes to cisplatin resistance in prostate cancer cells by modulating a specific anti-apoptotic pathway	Villar, J. et al. PCPH/ENTPD5 expression enhances the invasiveness of human prostate cancer cells by a protein kinase C delta-dependent mechanism (42).
	Hepatocellular Cancer	Depletion of ENTPD5 in mice leads to progressive hepatopathy.	Mice deficient in ENTPD5 developed progressive hepatopathy characterized by centrilobular hepatocyte hypertrophy, increased hepatocyte turnover, and hepatocellular neoplasia with age.	Read, R. et al. Ectonucleoside triphosphate diphosphohydrolase type 5 (Entpd5)-deficient mice develop progressive hepatopathy (14),
		Expression of ENTPD5 is related to HBV-associated hepatocarcinogenesis.	The analysis of HBV-associated hepatocarcinogenesis revealed that ENTPD5 is part of a long noncoding RNA–mediated competing endogenous RNA (ceRNA) network involving EHHADH-AS1 and hsa-miR-4459.	Wen, Q. et al. Competing endogenous RNA screening based on long noncoding RNA-messenger RNA co-expression profile in Hepatitis B virus-associated hepatocarcinogenesis (43).
		ENTPD5 is found overexpressed in HCC cells and KT/Ras-driven liver tumors in mice.	ENTPD5 is a downstream target of the mTORC1-regulated 4EBP1/eIF4E axis, with increased expression observed in AKT/Ras-driven liver tumors in mice and in human hepatocellular carcinoma (HCC) cells.	Wang, C. et al. 4EBP1/eIF4E and p70S6K/RPS6 axes play critical and distinct roles in hepatocarcinogenesis driven by AKT and N-Ras proto-oncogenes in mice (44).
	Colorectal Cancer	Expression of ENTPD5 is involved in increased resistance to oxaliplatin.	The expression of mt-PCPH in colorectal cancer cells significantly reduced intracellular ATP levels and increased resistance to the chemotherapeutic agent oxaliplatin.	MacCarthy, C. M. & Notario, V. The ENTPD5/mt-PCPH oncoprotein is a catalytically inactive member of the ectonucleoside triphosphate diphosphohydrolase family (45).
		ENTPD5 is decreasing in the progress from normal mucosa to adenoma and cancer.	ENTPD5 showed a continuous decrease pattern in expression from normal mucosa to adenoma and carcinoma.	Mikula, M. et al. Integrating proteomic and transcriptomic high-throughput surveys for search of new biomarkers of colon tumors (46).
	Non-Small Cell Lung Cancer (NSCLC)	Overexpression of ENTPD5 is related to poor prognosis.	ENTPD5 expression was elevated in lung cancer tissues and significantly associated with patient sex, smoking history, and tumor stage. Patients with negative ENTPD5 expression had longer overall survival.	Xue, Y. et al. ENTPD5 induces apoptosis in lung cancer cells via regulating caspase 3 expression (47).
		ENTPD5 knockdown led to increased apoptosis.	Gene expression profiling and molecular analysis revealed that ENTPD5 knockdown led to increased Caspase 3 expression, and the use of a Caspase 3 inhibitor partially reversed the induced apoptosis.	Xue, Y. et al. ENTPD5 induces apoptosis in lung cancer cells via regulating caspase 3 expression (47).
		Suppression of ENTPD5 enhanced tumor sensitivity to dietary restriction.	A Kras-driven, PTEN-deficient mouse model of lung cancer showed that coexisting tumors within the same lung exhibited marked differences in Akt signaling activity and sensitivity to dietary restriction.	Curry, N. L. et al. Pten-null tumors cohabiting the same lung display differential AKT activation and sensitivity to dietary restriction (32).
	p53-mutant cancers	Decreasing ENTPD5 or noted mutant p53 expression leads to decreased metastasis in breast cancer mouse models.	Mutant p53 increases ENTPD5 levels, which subsequently enhances the calnexin/calreticulin (CANX/CALR) chaperone cycle within the endoplasmic reticulum.	Vogiatzi, F. et al. Mutant p53 promotes tumor progression and metastasis by the endoplasmic reticulum UDPase ENTPD5 (29).
		Overexpression of ENTPD5 facilitates the metastasis of p53-mutant cancers.	mutp53 enhances ENTPD5 expression, which in turn maintains high protein levels of integrin-α5 (ITGA5) and integrin-β1 (ITGB1) through the CANX/CALR cycle.	Pavlakis, E. et al. Mutant p53-ENTPD5 control of the calnexin/calreticulin cycle: a druggable target for inhibiting integrin-α5-driven metastasis (6).
	PTEN-deficient cancers	Increased expression of ENTPD5 contributes to increased metastasis.	Depletion of PTEN in cancer cells led to increased ENTPD5 expression, which subsequently enhanced IGF1R signaling, leading to increased metastatic behavior.	Yu, Y. et al. PTEN phosphatase inhibits metastasis by negatively regulating the Entpd5/IGF1R pathway through ATF6 (48).
ENTPD6	Lung squamous cell carcinoma (LUSC)	ENTPD6 is overexpressed in high-risk LUSC patients.	ENTPD6 expression was elevated in the high-risk LUSC group and was significantly correlated with the infiltration levels of CD8+ T cells and B cells.	Zhuang, Z. & Gao, C. Development of a Clinical Prognostic Model for Metabolism-Related Genes in Squamous Lung Cancer and Correlation Analysis of Immune Microenvironment (49).
	Testicular cancer	Lower ENTPD6 level is related to cisplatin resistance in testicular cancer.	Level of ENTPD6 was significantly lower in cisplatin-resistant testicular cancer NEC-8/DDP cells compared to their parental NEC-8 cells.	Tada, Y. et al. Ectonucleoside triphosphate diphosphohydrolase 6 expression in testis and testicular cancer and its implication in cisplatin resistance (21)
	Lymphoma	Knockdown of ENTPD6 is related to dFdC and AraC resistance.	siRNA-mediated knockdown of *ENTPD6* decreased sensitivity to dFdC and AraC, indicating that lower *ENTPD6* expression may impair chemotherapeutic effects in human lymphoblastoid cell lines.	Fridley, B. L. et al. Gene set analysis of purine and pyrimidine antimetabolites cancer therapies (50).

#### 5.1.1 Potential pathways that mediates ENTPD5’s role in cancer

Vogiatzi et al. revealed ENTPD5 as a significant factor in the progression and metastasis of cancers driven by mutant p53. They have shown that mutant p53 increases ENTPD5 levels, which subsequently enhances the calnexin/calreticulin (CANX/CALR) chaperone cycle within the endoplasmic reticulum. This process ensures proper glycoprotein folding, such as that of integrin-α5, which is key to metastatic spread. Moreover, they observed that decreasing ENTPD5 or mutant p53 expression was associated with decreased metastases in breast cancer mouse models (29). Other than this study, other work conducted by Pavlakis et al. also demonstrated similar findings (6). Besides breast cancer mouse models, they observed this regulation across several p53 missense mutants and cancer types, including pancreatic and lung adenocarcinomas. Disruption of this axis impaired ITGA5-dependent adhesion, migration, and invasion without affecting cell proliferation. These findings indicate that the mutp53–ENTPD5–ITGA5 pathway may play a role in facilitating metastatic progression and suggest potential therapeutic targets.

In another study, Yu et al. have demonstrated that PTEN suppresses metastasis by downregulating the ENTPD5-IGF1R (insulin-like growth factor 1 receptor) pathway via ATF6 (activating transcription factor 6) (48). Depletion of PTEN in cancer cells was linked to increased ENTPD5 expression, which subsequently enhanced IGF1R signaling and contributed to increased metastatic behavior. This effect was mediated through ATF6 activation, which is a regulator of endoplasmic reticulum stress. Moreover, inhibiting the PTEN-ENTPD5-IGF1R pathway reduced tumor spread *in vitro* and *in vivo*, confirming the potential of targeting this pathway in PTEN-deficient cancers. These results highlight the significant role of PTEN in metastasis suppression.

#### 5.1.2 ENTPD5 in ovarian cancer

Chen et al. found that ENTPD5 expression was elevated in serous ovarian cancer tissues. Silencing ENTPD5 can result in reductions in tumor cell proliferation, migration, and invasion in mouse models (40). *ENTPD5* knockdown activated the GRP78 (glucose-regulated protein 78)/p-eIF-2α (phosphorylated eukaryotic translation initiation factor 2 alpha)/CHOP (C/EBP homologous protein) pathway, inducing ER stress and triggering cell apoptosis. Additionally, ENTPD5 expression was found to be closely correlated with clinical stage and patient prognosis. These results indicate that ENTPD5 may drive ovarian cancer progression by modulating ER stress-related signaling pathways.

In the study by Wang et al., multiple bioinformatics tools combined with clinical validation revealed that ENTPD5 was highly enriched in several pathways related to metabolism and signal transduction, including the WNT (Wingless/Int-1) and insulin signaling pathways (41). ENTPD5 expression was negatively correlated with the infiltration of NK cells, mast cells, and eosinophils, which suggests a possible role of ENTPD5 in modulating the immune microenvironment during tumor progression.

#### 5.1.3 ENTPD5 in prostate cancer

Villar et al. found that ENTPD5 is upregulated in various cancers, particularly in prostate cancer (42). Immunohistochemistry and transcriptomic data confirmed that high ENTPD5 expression is closely associated with poor prognosis in prostate cancer patients. Studies have also demonstrated that ENTPD5 promotes tumor cell growth and survival by enhancing protein glycosylation and folding efficiency. The study also revealed that ENTPD5 expression is regulated by the PTEN/PI3K/AKT signaling pathway, suggesting that it may act as a mediator of the oncogenic effects of this pathway in prostate cancer.

Villar et al. also found that expression of PCPH and its oncogenic variant mt-PCPH contributes to cisplatin resistance in prostate cancer cells by regulating a specific anti-apoptotic pathway (42). Knockdown of PCPH, mt-PCPH, or PKCα led to decreased phosphorylation and expression of Bcl-2, causing the cells to become more susceptible to cisplatin-induced apoptosis. Re-expression of Bcl-2 can restore resistance. These findings identify a functional PCPH–PKCα–Bcl-2 axis that enhances prostate cancer cell survival under chemotherapeutic stress.

Durst et al. used a high-throughput screening of over 20,000 compounds to identify small molecules that could inhibit ENTPD5 enzymatic. Through structure-activity relationship analysis, they identified two representative compounds. Further validation in prostate cancer cells showed that one of these compounds significantly reduced intracellular glycoprotein levels and suppressed cancer cell proliferation (51), providing an experimental foundation for further investigation into ENTPD5 as a potential anti-cancer target.

#### 5.1.4 ENTPD5 in hepatocellular cancer

Read et al. (2009) found that as Entpd5-deficient mice aged, the animals developed progressive centrilobular hepatocyte hypertrophy, increased hepatocyte turnover, and hepatocellular neoplasia (14). This study also described evidence of endoplasmic reticulum stress, accumulation of ubiquitinated proteins, and irregular protein glycosylation patterns in hepatocytes lacking ENTPD5, indicating possible involvement of disrupted protein processing pathways.

In a further study by Wen et al., analysis of HBV (Hepatitis B Virus)-associated hepatocarcinogenesis revealed that ENTPD5 is part of a long noncoding RNA–mediated competing endogenous RNA (ceRNA) network involving EHHADH (Enoyl-CoA Hydratase And 3-Hydroxyacyl CoA Dehydrogenase)-AS1(Antisense RNA 1) and hsa-miR-4459. The expression of ENTPD5 was positively correlated with that of EHHADH-AS1 (Pearson correlation coefficient > 0.9). In addition, compound K (20-O-β-D-glucopyranosyl-20(S)-protopanaxadiol) treatment blocked the down-regulation of EHHADH-AS1 in multiple liver cancer cell lines (43).

Additionally, Wang et al. found that ENTPD5 is a downstream target of the mTORC1-regulated 4EBP1/eIF4E (Eukaryotic Translation Initiation Factor 4E–Binding Protein 1/Eukaryotic Initiation Factor 4E) axis, with increased expression observed in AKT/Ras-driven liver tumors in mice and in HCC cells. Inhibition of eIF4E activity through the expression of 4EBP1A4 significantly reduced the levels of ENTPD5 and associated factors AK1(Adenylate Kinase 1) and CMPK1 (Cytidine Monophosphate Kinase 1), and delayed tumor development (44). Silencing of ENTPD5 in AKT (Protein Kinase B)/Ras tumor cells led to decreases in cell proliferation and increased apoptosis, while no major changes were observed in markers of endoplasmic reticulum stress or glycolytic enzymes, suggesting that ENTPD5 may have other context-dependent functions in HCC.

#### 5.1.5 ENTPD5 in colorectal cancer

MacCarthy et al. found that expression of mt-PCPH in colorectal cancer cells significantly decreased intracellular ATP levels and increased resistance to the chemotherapeutic agent oxaliplatin (45). Using a combination of *in vitro* and *in situ* assays, the authors have shown that the mt-PCPH protein lacks detectable NTPDase activity, indicating that the observed ATP depletion is unlikely to result from direct ATP hydrolysis. These findings strongly support that mt-PCPH is a structurally inactive member of the NTPDase family and suggest that it may influence cellular energy metabolism and chemotherapeutic response through mechanisms independent of the enzymatic activity.

Michal Mikula et al. studied gene expression microarray data and proteomic analysis to compare samples from normal colon mucosa, adenomas, and adenocarcinomas to find potential biomarkers involved in colon tumor development. They found 15 proteins with progressively increased expression and 23 with progressively decreased expression across the normal–adenoma–carcinoma sequence. By comparing proteomic and transcriptomic data and validating selected genes by qRT-PCR in independent samples, six genes were confirmed to exhibit stepwise expression changes during tumor progression. Among them, ENTPD5 showed a continuous decrease in expression from normal mucosa to adenoma and carcinoma (46).

#### 5.1.6 ENTPD5 in non-small cell lung cancer

Xue et al. investigated the expression of ENTPD5 in NSCLC and found that ENTPD5 expression was elevated in lung cancer tissues and significantly associated with patient sex, smoking history, and tumor stage. Patients with lower ENTPD5 expression had longer overall survival. The authors also found that knocking down ENTPD5 in A549 and PC9 cells significantly decreased cell proliferation, migration, and invasion, while increasing cell apoptosis. Additionally, they also demonstrated that ENTPD5 suppression inhibited tumor growth and facilitated apoptosis. ENTPD5 knockdown can lead to increased Caspase 3 expression, and the use of a Caspase 3 inhibitor could reverse the induced apoptosis (47).

In a study conducted by Curry et al., a Kras-driven, *PTEN*-deficient mouse model of lung cancer revealed that coexisting tumors within the same lung showed differences in AKT signaling activity and sensitivity to dietary restriction (DR). Specifically, bronchiolar-origin tumors showed high AKT activity and resistance to DR, while alveolar-origin tumors had low AKT activity and were sensitive to DR. Further investigation demonstrated that suppression of ENTPD5 can reduce IGF1R levels and enhance tumor sensitivity to DR (32).

In conclusion, ENTPD5 showed a complex expression pattern and functions in different types of tumors. Some studies demonstrated its oncogenic role in certain cancers, where it facilitates tumor cell survival and proliferation by enhancing protein folding and glycosylation. In contrast, in colorectal cancer, several studies—including those by Perilli et al. (52) and Mikula et al. (46)—have observed a consistent downregulation of ENTPD5 during tumor progression, suggesting a potential tumor-suppressive function.

Taken together, these results indicate that the physiological role of ENTPD5 may be tissue-specific. Therefore, it is necessary to further investigate the regulatory mechanisms of ENTPD5 expression, its functional roles in different tissues, and its interaction with different tumor microenvironments.

### 5.2 ENTPD6 in cancer: a potential purinergic modulator of drug sensitivity

Although ENTPD6 is not known to act as a proto-oncogene, this ectoenzyme may influence cancer progression and sensitivity to cancer therapy through modulation of purinergic signaling as well as subsequent AKT changes in the tumor microenvironment. Zifan Zhuang et al. have analyzed lung squamous cell carcinoma (LUSC) samples from the TCGA (The Cancer Genome Atlas) database and identified nine metabolism-related genes significantly associated with prognosis, including ENTPD6. Based on these genes, they developed a clinical prognostic model.

This study found that ENTPD6 expression was elevated in the high-risk group and was significantly correlated with the infiltration levels of CD8+ T cells and B cells (49). In addition, copy number variations of ENTPD6 were associated with immune cell infiltration. These findings indicate that ENTPD6 may play a role in modulating the tumor immune microenvironment in LUSC and has potential value in clinical prognostic assessment.

Tada et al. found that the expression level of ENTPD6 was significantly decreased in cisplatin-resistant testicular cancer NEC-8 (Neuroendocrine Carcinoma)/DDP (Cisplatin) cells compared to their parental NEC-8 cells (21). This finding was also observed in other cisplatin-resistant bladder cancer cells. Further investigation showed that ENTPD6 expression was higher in normal tissue than in cancer tissues, and its expression in seminoma is higher than in other types of testicular tumors. In addition, ENTPD6 was found to interact with E-cadherin and regulate its expression, thereby influencing the tumor sensitivity to cisplatin. These results suggest that ENTPD6 may serve as a potential molecular marker and therapeutic target for cisplatin resistance in testicular cancer.

Additionally, in a study conducted by Fridley et al., the authors systematically evaluated the relationship between baseline mRNA expression levels and cytotoxicity of four antimetabolite anticancer drugs—specifically, the pyrimidines dFdC (Gemcitabine) and AraC (Cytarabine), and the purines 6-TG (6-Thioguanine) and 6-MP (6-Mercaptopurine)—using human lymphoblastoid cell lines through gene set analysis. The study identified that the gene set nucleoside-diphosphatase activity was significantly associated with both dFdC and AraC, while the gamma-aminobutyric acid catabolic process was significantly associated with dFdC and 6-TG.

Further functional validation experiments demonstrated that four genes selected from the significantly associated pyrimidine-related gene sets, including *ENTPD6*, were successfully validated in SU86 cells. siRNA-mediated knockdown of *ENTPD6* led to reduced sensitivity to dFdC and AraC, indicating that decreased ENTPD6 expression may impair the efficacy of these drugs (50).

## 6 Clinical implications and future perspectives

### 6.1 Clinical applications of targeting ENTPD5

ENTPD5 plays multiple roles in cancer development and progression, making it a potential target for cancer therapy. As a UDPase located in the endoplasmic reticulum, ENTPD5 facilitates glycoprotein folding and secretion. This function is especially important in tumor cells with high secretory activity. Inhibiting this enzymatic activity may influence protein processing, induce endoplasmic reticulum stress, and promote apoptosis. Although no ENTPD5 inhibitors are currently in clinical use, high-throughput screening is possibly needed to identify potential small-molecule candidates.

Some AKT and mTOR inhibitors, such as rapamycin and everolimus, and some PI3K inhibitors, may reduce the expression of ENTPD5. Thus, in some tumors with PTEN- or AKT-mutant, combining these inhibitors with the ENTPD5 targeting strategy may enhance the therapeutic efficacy. In addition, the role of ENTPD5 in stabilizing ER function also provides a rationale for combination strategies with ER stress-inducing agents. Combined with ENTPD5 suppression, these agents may exceed the stress tolerance of tumor cells and trigger cell death. ENTPD5 also affects the tumor microenvironment by regulating glycoprotein secretion, including proteins involved in immune response. This activity may contribute to immune evasion. As a result, ENTPD5 inhibition has the potential to improve the efficacy of immune checkpoint inhibitors, such as anti-PD-1(Programmed Cell Death Protein 1) or anti-PD-L1(Programmed Death-Ligand 1)antibodies. Therapeutic strategies targeting mutant p53-ENTPD5 are also promising, including integrin antibodies and α-glucosidase inhibitors (6), and so on. For example, HSP90(Heat Shock Protein 90) inhibitors can decrease mutant p53, which can suppress cancer cell metastasis. These studies highlight the potential of targeting ENTPD5 in cancer treatment, either by directly inhibiting its activity or disrupting its associated regulatory pathways.

In a study conducted by Perilli et al., they found that expression of miR-182 increases significantly in the development of colorectal cancer, including in normal mucosa, adenomas, primary tumors, and liver metastases. Concurrently, ENTPD5 also shows downregulation at both the mRNA and protein levels (52). This study also showed that miR-182 could target the 3′ untranslated region (UTR) of ENTPD5. In addition, miR-182 is significantly higher in the plasma of colorectal cancer patients when compared to healthy controls and polyp patients, and is significantly decreased after surgical removal of liver metastases. In contrast, miR-215 specifically targets the alternative terminal exon, known as exon 19, of ENTPD5 transcripts. These findings suggest that miR-182 and miR-215, both regulating ENTPD5 through distinct mechanisms, may play important roles in cancer development (4).

Moreover, ENTPD5 was reported to be a downstream mRNA target of miR-28 in the nervous system, indicating that miR-28–mediated post-transcriptional regulation may represent another potential mechanism and strategy controlling ENTPD5 expression (53). Among these, the regulation by miR-182 currently stands as the most well-documented, underscoring its particular potential as a non-invasive blood biomarker for disease monitoring.

In conclusion, ENTPD5 is involved in several important processes, including protein folding, metabolic regulation, and immune response, making this a promising target for targeted therapy. The major obstacle is the experimental work showing paradoxical development of HCC in mice globally null for ENTPD5 (14). Future research should focus on developing safe, selective inhibitors, investigating potential combination treatments to further facilitate translational clinical application.

### 6.2 Clinical applications of targeting ENTPD6

Currently, there are no well-characterized, validated small-molecule inhibitors for ENTPD5 or ENTPD6 that have been studied in preclinical models. A preliminary study within patent disclosure WO2012065139A2 (https://patents.google.com/patent/WO2012065139A2/en) has proposed potential ENTPD5-targeting compounds derived from natural products, including sesquiterpenoid hydroquinone and quinone derivatives. These compounds exhibited ENTPD5 inhibition *in vitro* and may promote cancer cell apoptosis or enhance sensitivity to chemotherapy. However, their biological activity and specificity remain largely untested.

Still, ENTPD5/6 do remain promising therapeutic targets because of their likely roles in maintaining nucleotide sugar homeostasis and regulating protein N-glycosylation. These are critical for cancer cell survival and proliferation. Future development of selective inhibitors will be essential to fully evaluate the therapeutic potential of targeting these enzymes.

Although ENTPD6 has been less well studied, existing research suggests that it participates in NDPs metabolism, and potentially protein glycosylation, which may further influence tumor growth, drug-resistance, and metastasis. ENTPD6 hydrolyzes extracellular NDPs, thereby affecting nucleotide levels that regulate cancer cell proliferation and immune evasion. In preclinical studies, P2 receptor antagonists (such as P2X7 and P2Y inhibitors (54)) have been applied to disrupt purinergic signaling in glioblastoma (55) and breast cancer (56). These strategies offer indirect references and support for modulating ENTPD6-related pathways.

Since extracellular ATP and ADP facilitate immune activation but can be potentially influenced by ENTPD6, suppressing ENTPD6 activity may help increase nucleotide accumulation. This could stimulate immune responses. In addition, as adenosine accumulation can suppress immune responses, combining ENTPD6 inhibition with immune checkpoint inhibitors, such as anti-PD-1 or anti-PD-L1 antibodies, may improve their efficacy. Similar approaches have been considered in studies involving CD39 (10).

ENTPD5 is a potential target for cancer therapy due to its oncogenic role in many tumor types, such as facilitating glycoprotein folding, supporting secretory activity, and maintaining ER homeostasis. In contrast, ENTPD6 still needs more studies to unveil its role in cancer development. The main function is to regulate UDP metabolism, which may further influence protein glycosylation and cancer progression. Several questions remain to be studied, for example, the potential role of ENTPD6 in different kinds of cancer types, the interaction with other purinergic enzymes such as CD39, and the critical role it may play in protein glycosylation, and so on.

## 7 Conclusion: ENTPD5 and ENTPD6

### 7.1 Comparison of ENTPD5 and ENTPD6

ENTPD5 and ENTPD6 are both closely related members of the same ectonucleotidase family, but the respective cellular localizations and biological functions are quite different. ENTPD5 is mainly localized in the endoplasmic reticulum. It can hydrolyze UDP to maintain the nucleotide sugar balance required for protein N-glycosylation. This activity facilitates proper protein folding and reduces ER stress, particularly in cancer cells, for example, with hyperactivated PI3K/AKT signaling, thereby promoting tumor growth and metastasis. Consistent with these functions, ENTPD5 has been described as an oncogene in multiple cancer contexts.

In contrast, ENTPD6 has been less well characterized but seems expressed in the Golgi apparatus. Biochemical studies indicate that this ectoenzyme hydrolyzes mainly UDP and GDP, thereby participating in extracellular/luminal nucleotide metabolism and membrane trafficking. Hence, ENTPD6 may influence glycosylation and metabolism pathways. It is also flagged in the Human Protein Atlas as a “cancer-related gene,” though with relatively low specificity compared to classical oncogenes. Additionally, the ENTPD6 functionality has been associated with chemotherapy resistance.

### 7.2 Implications for therapeutic targeting

These functional differences between these two ectonucleotidases highlight distinct therapeutic opportunities. For ENTPD5, preliminary efforts have started to explore small-molecule inhibitors. As an example, within the patent WO2012065139A2, the authors describe natural product–derived compounds, such as sesquiterpenoid hydroquinone and quinone derivatives, that inhibit ENTPD5 activity *in vitro* and may sensitize cancer cells to chemotherapy. However, no well-characterized inhibitors have been validated in preclinical models or in clinical trials. More to the point, ENTPD6 currently lacks any reported inhibitors. These issues underscore the need for further drug discovery efforts targeting these enzymes.

ENTPD5 and ENTPD6 may also contribute to immune regulation and metabolic disorders. ENTPD5-driven glycosylation supports tumor immune evasion, while ENTPD6 has been potentially associated with modulation of purinergic signaling and potentially T-cell responses. In addition, because both enzymes regulate nucleotide sugar balance, their dysregulation may be relevant to metabolic syndromes and glycosylation-related diseases. Future studies focusing on these aspects will also be critical for understanding the full spectrum of ENTPD5/6 biology and any future therapeutic application.

## Glossary

4EBP1: Eukaryotic Translation Initiation Factor 4E–Binding Protein 1
6-MP: 6-Mercaptopurine
6-TG: 6-Thioguanine
ADM: Adrenomedullin
ADP: Adenosine diphosphate
AK1: Adenylate Kinase 1
AKT: Protein Kinase B (PKB)
ALT: Alanine aminotransferase
AraC: Cytarabine
AS1: Antisense RNA 1
ATF6: Activating transcription factor 6
ATP: Adenosine triphosphate
BAT: Brown adipose tissue
BMI: Body mass index
CALR: Calreticulin
CANX: Calnexin
CD39: Cluster of Differentiation 39 (ENTPD1)
CD73: Cluster of Differentiation 73/Ecto-5′-Nucleotidase
CDP: Cytidine diphosphate
CHOP: C/EBP homologous protein
CMPK1: Cytidine Monophosphate Kinase 1
CRC: Colorectal cancer
CTP: Cytidine triphosphate
CYP1A: Cytochrome P450 family 1 subfamily A
CYP3A: Cytochrome P450 family 3 subfamily A
DDP: Cisplatin
dFdC: Gemcitabine
DMBA: Dimethylbenzanthracene
DR: Dietary restriction
EHHADH: Enoyl-CoA Hydratase And 3-Hydroxyacyl CoA Dehydrogenase
eIF4E: Eukaryotic Initiation Factor 4E
ENPP1: Ectonucleotide pyrophosphatase phosphodiesterase 1
ENTPD: Ectonucleoside triphosphate diphosphohydrolase
ENTPD1: Ectonucleoside triphosphate diphosphohydrolase 1
ENTPD3: Ectonucleoside triphosphate diphosphohydrolase 3
ENTPD5: Ectonucleoside triphosphate diphosphohydrolase 5 (PCPH)
ENTPD6: Ectonucleoside triphosphate diphosphohydrolase 6
ER: Endoplasmic reticulum
GDP: Guanosine diphosphate
GRP78: Glucose-regulated protein 78
GTP: Guanosine triphosphate
GWAS: Genome-wide association studies
HBV: Hepatitis B Virus
HCC: Hepatocellular carcinoma
HFHC: High-fat, high-cholesterol diet
HNSC: Head and neck squamous cell carcinoma
HRD1: HMG-CoA Reductase Degradation Protein 1
HSP90: Heat Shock Protein 90
IDP: Inosine diphosphate
IGF1R: Insulin-like growth factor 1 receptor
ITGA5: Integrin subunit alpha 5
LUSC: Lung squamous cell carcinoma
MAPK: Mitogen-activated protein kinase
MASH: Metabolic dysfunction-associated steatohepatitis
MASLD: Metabolic dysfunction-associated steatotic liver disease
MGI: Mouse Genome Informatics
mTOR: mechanistic Target of Rapamycin
NAFLD: Non-alcoholic fatty liver disease
NEC: Neuroendocrine Carcinoma
NK: Natural Killer Cell
NSCLC: Non-Small Cell Lung Cancer
PCOS: Polycystic ovary syndrome
PCPH: Prostate Cancer Progression-Associated Protein/ENTPD5
PCR: Polymerase chain reaction
PD-1: Programmed Cell Death Protein 1)
PDAC: Pancreatic ductal adenocarcinoma
PD-L1: Programmed Death-Ligand 1
PI3K: Phosphoinositide 3-kinase
PTEN: Phosphatase and tensin homolog
RNA: Ribonucleic Acid
RPS6: Ribosomal Protein S6
TCGA: The Cancer Genome Atlas
UDP: Uridine diphosphate
UDPase: Uridine diphosphatase
UMP: Uridine monophosphate
UTP: Uridine triphosphate
UTR: Untranslated region
WNT: Wingless/Int-1.
